# Security Requirements and Challenges of 6G Technologies and Applications

**DOI:** 10.3390/s22051969

**Published:** 2022-03-02

**Authors:** Shimaa A. Abdel Hakeem, Hanan H. Hussein, HyungWon Kim

**Affiliations:** 1 School of Electronics Engineering, Chungbuk National University, Cheongju 28644, Korea; shimaakotb@cbnu.ac.kr; 2 Electronics Research Institute (ERI), El Nozha, Cairo 12622, Egypt; hananhussein@eri.sci.eg

**Keywords:** 6G security, privacy, new challenges, security architecture, security threats, physical layer security, AI/ML security

## Abstract

After implementing 5G technology, academia and industry started researching 6th generation wireless network technology (6G). 6G is expected to be implemented around the year 2030. It will offer a significant experience for everyone by enabling hyper-connectivity between people and everything. In addition, it is expected to extend mobile communication possibilities where earlier generations could not have developed. Several potential technologies are predicted to serve as the foundation of 6G networks. These include upcoming and current technologies such as post-quantum cryptography, artificial intelligence (AI), machine learning (ML), enhanced edge computing, molecular communication, THz, visible light communication (VLC), and distributed ledger (DL) technologies such as blockchain. From a security and privacy perspective, these developments need a reconsideration of prior security traditional methods. New novel authentication, encryption, access control, communication, and malicious activity detection must satisfy the higher significant requirements of future networks. In addition, new security approaches are necessary to ensure trustworthiness and privacy. This paper provides insights into the critical problems and difficulties related to the security, privacy, and trust issues of 6G networks. Moreover, the standard technologies and security challenges per each technology are clarified. This paper introduces the 6G security architecture and improvements over the 5G architecture. We also introduce the security issues and challenges of the 6G physical layer. In addition, the AI/ML layers and the proposed security solution in each layer are studied. The paper summarizes the security evolution in legacy mobile networks and concludes with their security problems and the most essential 6G application services and their security requirements. Finally, this paper provides a complete discussion of 6G networks’ trustworthiness and solutions.

## 1. Introduction

By 2020, fifth-generation (5G) radio networks had been implemented globally, with features such as mass connection, extreme dependability, and guaranteed low latency specified [1]. 5G, on the other hand, will fall short of meeting all future needs beyond 2030. Sixth generation (6G) wireless network technology is predicted to offer higher coverage, less energy consumption, comprehensive spectral, and cost-effectiveness with improved security. 6G networks will meet these needs by deploying new technologies such as multiple accesses, waveform design, channel coding schemes, network slicing, numerous antenna technologies, and cloud edge computing. 6G affects four significant future changes [2]. First, it offers an integrated air–ground–space–sea communication network by deploying terrestrial and non-terrestrial networks [3]. Second, new radio bands will improve network traffic capacity and data speed, including millimeter-wave (mm-wave), sub-6 GHz, terahertz (THz), and optical communications. Third, 6G will enable a new generation of intelligent applications and services using artificial intelligence (AI) and big data technologies in response to the massive datasets generated by heterogeneous networks with different communication scenarios, wide bandwidths, a higher number of antennas, and new 6G applications’ requirements [4,5,6,7]. Fourth, network security and privacy must be strengthened and enhanced for 6G technologies and applications [8]. This paper presents the 6G security trends and challenges of other upcoming technologies and applications. Data processing, threat detection, traffic analysis, and data encryption are considered the most critical issues in 6G networks. The security issues due to massive traffic processing can be solved using decentralized security systems, in which the traffic can be handled dynamically and locally. 6G use cases impose stricter security requirements than 5G use cases [9,10]. The Internet of Everything (IoE), with a wide variety of capabilities and services, will make it more challenging to operate and install distributed AI, privacy, and security solutions. The high mobility conditions of the new connected devices make them change their interconnected networks and require services from other networks, resulting in security complications and privacy problems.

**Figure 1:**
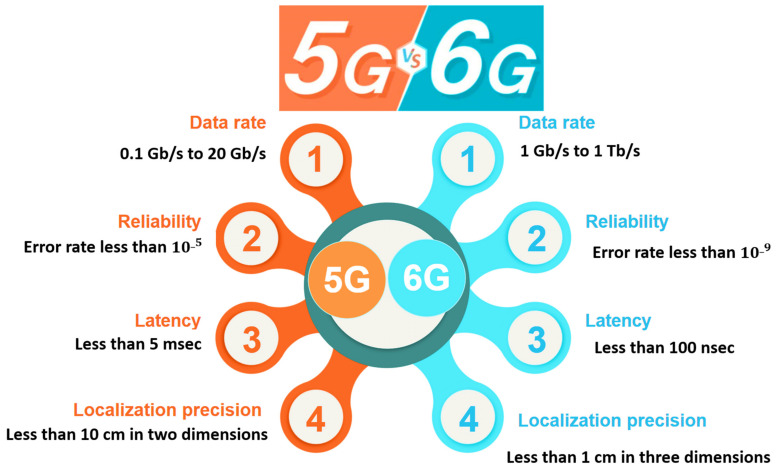
The 5G and 6G features comparison.

For the Enhanced Ultra-Reliable and Low Latency Communication (ERLLC) services, the end to end latency in 6G should be decreased to a few µs. Additionally, 6G will need a ten-time increase in network energy efficiency over 5G and a hundred-time increase over 4G [11]. It is predicted to allow very low-power transmissions for limited resource devices. Advanced and active management technologies for high mobility will enable rapid movement at 1000 km per hour. To guarantee the quality of the service for ERLLC, the latency effect of security processes will be evaluated. Similarly, high requirements need highly efficient security solutions that ensure service and resource availability. The IoE provides difficulties in deploying and operating the new distributed intelligent AI and ML security techniques. A critical element is figuring out how to incorporate new security enablers into resource-constrained devices [12]. Figure 1 summarizes the comparison between 5G and 6G in data rates, reliability, latency, and localization accuracy.

10^−9^ A comprehensive survey on security and privacy concerns with 6G networks is highlighted in this study. We briefly introduce the security development of the previous mobile radio generations (1G to 5G), focusing on the security shortcomings mentioned in existing solutions. The 6G security problems in different critical fields are investigated. Moreover, the study presents the 6G technologies and applications’ security issues and requirements. Then, we propose solutions for the emerging 6G applications. This paper considers one of the first studies that includes an extensive survey for the 6G new technology security potential solutions [13,14].

We summarize the paper contributions as follows:

1. Introducing the security issues in the earlier legacy mobile networks.
2. Presenting the 5G security architecture improvements and their effect on the new architecture of 6G.
3. Presenting the trending 6G technologies and studying the security requirements of each technology.
4. Studying the 6G applications and services requirements.
5. Presenting the 6G applications security problems and proposed solutions.

The rest of this paper is organized as follows. Section 2 shows the security issues and architecture development of legacy mobile networks. Section 3 introduces the 6G network vision and essential research projects. Section 4 introduces the security requirements of the proposed 6G architecture. 6G technologies’ security issues and possible solutions are presented in Section 5. Section 6 provides future security challenges and problems of 6G applications. The study is concluded in Section 7.

## 2. Security Evolution of Mobile Cellular Networks

This section discusses different cellular network generations’ security threats and privacy concerns. The early mobile generations encountered challenging security concerns, involving eavesdropping attacks, encryption issues, physical attacks, and authentication problems. Thus, the threat landscape has grown with more complex attacks and more competent attackers.

### 2.1. Security Issues in 1G, 2G, and 3G

In the 1980s, the 1G network was created specifically to deliver voice communications services. It uses analog modulation techniques to transfer data. This generation has several issues, involving handover problems, no guarantees on security, and many transmission concerns. In addition, due to the unencrypted nature of telephone services, data transmission cannot be guaranteed to be secure or private. As a result, the whole network and its users are exposed to significant security threats, including unauthorized access and eavesdropping attacks [15].

The second mobile generation depends on digital modulation protocols such as Time Division Multiple Access (TDMA) to enable voice and short messaging services. The GSM (Global System for Mobile Communications) standard [16] offers security services such as authentication, privacy protection, transmission protection, and personal information protection. Network providers use authentication to identify and authorize users [17]. The 2G authentication technique is based on a challenges and responses approach. Anonymity is achieved via anonymous identifiers that make it impossible to trace their actual identities. Encryption protects user data and signaling, while the SIM creates the encryption keys. Users save their privacy using Temporary Mobile Subscriber Identity (TMSI) and radio path encryption [18]. Unfortunately, despite considerable security advancements over the previous generation, there is still much vulnerability in 2G security. The one-way authentication issue is the security weaknesses in which the network can authenticate the user, but the user cannot be authenticated against the network [19]. As a result, unauthorized base stations work as legitimate members to steal users’ data and private information.

Furthermore, the end to end encryption problem occurs when a single part of the communication channel is encrypted. At the same time, the other network parts are unencrypted, which exposes the channel to attacks. Therefore, the mentioned TMSI privacy solutions and radio path encryption are insufficient to protect 2G networks and are susceptible to various attacks, including eavesdropping [20].

The 3G network was introduced in 2000 to increase the data transmission speed up to 2 Mbps and provide internet access. However, advanced services such as TV streaming, internet browsing, and video streaming are accessible at this speed, which is not feasible on the previous mobile communication [21]. 2G technology security is used to protect the 3G networks. Additionally, 3G addresses a number of the security vulnerabilities present in 2G. 3G includes two-way authentication and the Authentication and Key Agreement (AKA) [22]. The Third Generation Partnership Project (3GPP) establishes a complete access control security system, including air interface security and user authentication. The security of the air interface is used to protect communications over wireless links and users. At the same time, it provides a two-way authentication process that can authenticate users and the network on both sides (sender and receiver) for more reliability [23].

The 3GPP supports various privacy considerations for 3G networks, including securely locating, identifying, and tracking users. Internet Protocol (IP) vulnerabilities and attacks are considered a threat to 3G networks [24]. The communication channel attacks between the end devices and their home networks also introduce 3G network threats. The wireless interface threats are categorized into the following: (1) integrity threats, (2) unauthorized data access, (3) denial of service (DoS) attacks, and (4) unauthorized service access. AKA protocol privacy issues related to sniffing the users’ private information and identities are also considered critical security problems in 3G.

### 2.2. Security Issues in 4G and 5G

In 2009, 4G networks offered up to 1 Gbit per second for downlink transmission and 500 Mbit per second for uplink communication [25]. 4G networks also provide high spectrum efficiency and lower latency, enabling 4G networks to handle complex applications such as High-Definition Television (HD TV) and Digital Video Broadcasting (DVB). 4G systems include IP core networks, backbone, access networks, and a diversity of intelligent mobile terminals. The 4G primary security problems are related to threats of wireless radio communication, tampering, eavesdropping, data alteration, and network authentication. Due to the increased indirect interaction between users and mobile terminals, the 4G network is more vulnerable to security issues than previous mobile radio networks. Many security concerns incur severe damage with mobile terminal devices’ storage and computing improvements. Tampering hardware platforms, viruses, and operating system attacks are all security issues examples. The 4G standards and critical management protocols face different Medium Access Control (MAC) layer vulnerabilities, including eavesdropping and replay attacks. 4G networks are also vulnerable to data integrity attacks, problems of unauthorized users, and location tracking using the MAC layer protocols [26,27,28,29,30].

As the 5G network approaches commercialization, we may expect increased data speeds using complex systems and high-security architectures [31]. 5G networks’ novelty is their capacity to connect the growing number of devices while delivering higher quality services to all network entities. The most straightforward approach to categorize security and privacy issues in 5G networks is to examine the network architecture. The 5G architecture includes access networks, backhaul networks, and core networks. Many devices and network access methods present additional security issues. In addition, the handovers between different access technologies and different device types increase the probability of an attack [32,33,34,35,36].

Backhaul networks exist between the access and core networks through microwave connections, wireless channels, satellite links, and traditional lines. Because the backhaul networks lack devices’ connections, they pose fewer privacy problems than access networks. Additionally, security concerns are conveyed to the core network by moving the backhaul network into the data plane through techniques such as Software-Defined Networking (SDN) and Network Functions Virtualization (NFV) [37,38]. High data rates of Further Enhanced Mobile Broadband (FeMBB) pose security difficulties in the traffic probability of a DoS or resource attacks. Two methods for dealing with signaling overloads have been developed so far. The first method allows communication between many devices via lightweight authentication and key management techniques, while the second employs protocols that would enable the grouping of devices through many group-based AKA protocols. However, the new methods for accelerating the 5G network’s speed also create security vulnerabilities. For example, large MIMOs are utilized to hide active and passive eavesdropping. In addition, SDN implementation through OpenFlow poses a threat presented by rogue applications or activities.

**Figure 2:**
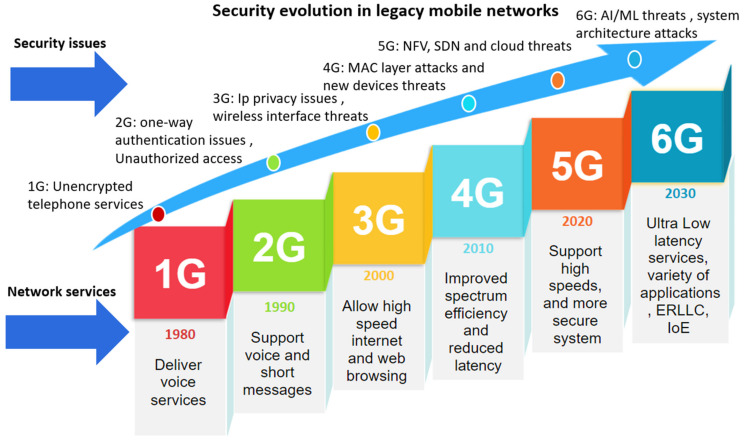
The security evolution of mobile communications from 1G to the predicted future 6G.

Moreover, NFV services migrate from one resource to another, presenting security issues. There are additional privacy issues related to many application scenarios and services types that 5G networks enable. Due to the 5G platform’s open nature, users’ private information is easily disclosed to the open state [39,40]. The privacy issues connected with 5G will undoubtedly become a problem in the future years that must be addressed and solved [41]. The CN of 5G consists of different functionalities. Networks are becoming more dynamic than ever due to NFV, SDN, and cloud technologies, resulting in many threats and vulnerabilities. The more devices and services that exceed the signaling load, the higher the new 6G applications’ criteria and greater network capacity than presently established 5G networks will be [42]. New 6G applications will have more criteria and need greater network capacity than presently established 5G networks [42]. Additionally, they have a significant impact on 6G operations. Therefore, security measures guarantee service continuity and quality in ERLLC [43]. Additionally, the latency effect due to security processes will be addressed. Effective security solutions are considered high requirements to ensure service and resources’ availability and continuity. Figure 2 summarizes the evolution and security issues from 1G to 6G.

### 2.3. 5G Security Improvements

5G improves security architecture and authentication methods while addressing many 4G flaws. 5G is the first standard to use unified authentication. WiFi, cable, and 3GPP networks are all supported. A 3GPP-authenticated UE may relocate to a non-3GPP network without reauthenticating [44]. 5G employs Subscription Concealed Identifier (SUCI) during authentication, an encrypted variant of Subscription Permanent Identifier (SUPI) [45]. Consequently, unencrypted data such as IMSI will not be sent across 5G networks. This feature increases network security. It also helps approve interceptions. Operators may intercept conversations for authorized law enforcement agents when a judge issues a subpoena to investigate a crime. However, the message format and entity role are different. RFC 5216 specifies EAP-TLS for IoT and private networks. Previously, non-USIM devices such as laptops or IoT devices could not subscribe or access the 5G core through EAP-TLS. 5G’s flaws and 5G’s complexity creates security problems. AKA fails to meet crucial goals in 5G. For example, the channel between the serving and home networks is not bounded. An attacker might use this issue to charge another user for network access. However, synchronization failure signals may be used to monitor users in 5G even if 5G–AKA overcomes IMSI-catcher attacks [46,47]. Another study [48] recommends utilizing paging to discover users with fewer than ten calls. Misleading a UE into revealing its SUPI is delivering a bogus pre-authentication message.

### 2.4. Conclusions of Mobile Networks Security

Every network generation has flaws. Although various measures to reduce exploitation exist, the difficulty of upgrading basic protocols leaves much vulnerability. Table 1 highlights the supported services, functions, and known security issues in the earlier generation security architectures. Attacks against 6G security architecture and applications include signaling DoS (denial of service), DDoS (dispersed denial of service) against authentication servers, energy depletion attacks, and user tracking. For example, poor authentication and resource restrictions affect all network generations and are difficult to perfect.

Following are the significant issues learned from legacy network security challenges and improvements.

- The security of new applications is usually compromised. Modern network standards outperform older network standards in new applications. However, they may introduce additional risks. Several studies projected these emerging apps’ vulnerability to impersonation and DoS attacks [49,50,51].
- Improving technology security before deployment is crucial. Support for an old protocol by a new protocol may reveal flaws. The fundamental cause is the incompatibility of two network security standards.
- Compatibility is frequently circumvented by requesting outdated architecture authentication. This access control method may reveal previous issues. Unwanted downgrades [52,53,54] push 4G-LTE devices onto old networks. Based on the absence of mutual verification between UE and authentication servers in 2G/3G standards, the attacker may then access the UE’s IMSI. It should be noted that dual network access authentication and identity management are security problems for 6G. More changes in protocol implementations than protocol designs decrease new vulnerabilities while improving vulnerability repairs.
- Large-scale essential equipment upgrades are necessary for AKA and subscriber identity management. Many operators and consumers may be financially impacted. Extensive security testing is required before implementing a new architectural or protocol design. Implementing protocol security patches or upgrading intrusion prevention systems at endpoints is feasible.
- A long-term design change is still necessary to fix the present architecture’s flaws and weaknesses.
- Mutual authentication and end to end encryption remain unsolved issues. Lack of these two properties causes false operators, eavesdropping, and tracing attacks. Due to high computational and communication demands, 5G is unlikely to meet these security standards. Encryption and mutual authentication in 6G may damage latency-sensitive services.

Resolving the present security vulnerabilities may become impossible if 6G is delayed.

## 3. 6G Network Vision and Essential Research Projects

This section discusses the network vision about the security architecture of 6G and the 6G initial supported projects’ requirements.

### 3.1. 6G Network Vision

5G technologies, including Multi-access Edge Computing (MEC), SDN, NFV, and network slicing, are still relevant to 6G networks. Therefore, their associated security matters will stay. For example, the most severe security concerns connected with SDN include vulnerabilities on the SDN controller, interfaces, and SDN applications platforms. Security obstacles associated with NFV include attacks on virtual machines, hypervisors, and virtual network function (VNF) managers. Finally, MEC is vulnerable to physical risks, DDoS, and the enormously distributed structure of 6G systems [55].

Information theft and DoS attacks through 6G network slices are possible network slicing attacks. Attacks against network automation technologies expose the 6G network’s capability to achieve high dynamicity and comprehensive network automation. 6G predicts that the IoE will become a reality involving billions of complex connected devices. The device’s primary security based on SIM cards is unsuitable for IoE deployment in 6G as 6G devices will be smaller than previous devices, such as in-body sensors. The required distribution and administration tasks are very inefficient in such an extensive network. Because IoT devices with constrained resources cannot guarantee complex encryption, they are a prime target for attackers. These tiny devices may be hacked and used to launch attacks. In addition, the data collected by intermediate IoE to support 6G applications create privacy concerns. Data theft through resource-constrained IoE devices harms data privacy. Local 5G network installations often focus on vertical markets such as industry, healthcare, and education. 6G extends the idea further by enabling even smaller networks such as in-body networks, drone swarms, and environmental sensor networks with increased battery life. These small networks function independently to communicate with wide area networks.

In contrast to the local 5G networks, many industries’ enablers support 6G with varying embedded security levels. 6G network with poor protection offers a chance for attackers to originate attacks. 6G cells will be decreased from small to tiny with high-density deployment. Device-to-Device (D2D) communications and mesh networks with multi-connectivity will become the 6G deployment standard. Malicious devices have a greater chance of attacking a dispersed network with more susceptible devices connected through the mesh, thus expanding the danger surface. The vast area network cannot provide security for the tremendous number of devices inside each sub-network [56].

In 6G, a hierarchical security mechanism that differentiates communication security at the sub-network level from sub-network to comprehensive area network security would be preferable. Convergence of the RAN and core functions centralizes the upper layer RAN services, synchronizing with scattered core functions such as User Plane Micro Services (UPMS) and Control Plane Micro Services (CPMS). Attackers may target UPMS and CPMS, impacting numerous radio units serviced by microservices. 6G networks include zero-touch networking and Service Management (ZSM) architecture to allow rapid services, low operating costs, and less human error. Complete automation combined with self-learning enables attacks to grow in closed-loop systems. Data privacy protection is challenging in zero-touch networks due to critical automation requirements with little human involvement [57,58].

### 3.2. The 6G Essential Projects

These days, the primary goal of all initiatives is to draw out long-term strategic roadmaps for the 6G wireless network. According to [59], more than EUR 95 million will be invested in B5G and 6G research between 2017 and 2025. These initiatives are supported by Horizon2020, the EU’s research and innovation framework program. Moreover, most of them are still in their infancy. Our focus in this part is on a few of these experiments and what they have learned about 6G security.

- Hexa-x

In 2021, the Hexa-x project was launched by Ericsson [60]. Different research institutions and universities are cooperating to commercialize the latest technologies in this project. The Hexa-x project aims to lay the basis of the 6G networks. It also aims to lead the research and Innovation (R&I) worldwide into the next generation. This project aims to improve tools essential to carry 6G networks to Europe. It will present new strategies to face six challenges: connecting intelligence, a network of networks, sustainability, global service coverage, trustworthiness, and extreme experience. Hexa-x will develop several axes to focus on these challenges [60]. New technologies such as AI and ML must be applied in communication among humans and devices to improve connection quality. The global digital ecosystem needs to create a single network of networks. This network should be heterogeneous, intelligent, and flexible. Resources should be exploited efficiently for a sustainable network. Affordable and practical solutions should be developed to support global and complete coverage for the 6G network. For high security, the next generation should assure data privacy, the integrity of communications, confidentially, and operational resilience. In addition, several technologies will be developed, such as network architecture, AI-driven air interface, THz radio access, and network virtualization to enhance the performance of 6G. The project will work on these groundbreaking communication technologies to link the physical, digital, and human worlds closer together.

- RISE 6G

RISE 6G (Reconfigurable Intelligent Sustainable Environments for 6G wireless networks) is one of the significant projects launched in 2021 [61]. The project exploits Reconfigurable Intelligent Surfaces (RIS) technology. RIS will become one of the powerful developing technologies in the future. RIS deals with the dynamicity of radio wave propagation control. It allows the perception of the wireless environment as a service. RISE 6G seeks to improve 6G capabilities for a sustainable, flexible, and intelligent wireless environment by exploiting RIS. The project will face four challenges related to RIS [62]. First, the actual RIS-assisted signal propagation will be modeled. Second, the new network architecture will be merged with multiple RISs. Third, several use cases will be designed to empower QoS, such as precision localization, green communication, power consumption, and massive capacity in a dynamic wireless programmable environment. Fourth, a prototype benchmark will be recommended for novelty based on two complementary proceedings. The project participates in standardization and brings its technical vision into the industrial implementation [63].

- New-6G

NEW-6G project will concentrate on the nano-world. The project links “microelectronic with telecom, network with equipment, and software with hardware.” Essentially, the project will develop new strategies and technologies to raise the network performance, such as [64]:

1. Network architecture and optimization.
2. Protocols and data flow.
3. Security of information and infrastructures.
4. Integrated circuits, digital components, high-performance radio frequencies, and low energy consumption.
5. Dedicated, high-performance, and sustainable semiconductor technologies.
6. New mechanisms will be offered by NEW-6G to exploit nano-electronics technology. Nano-electronics technology will be explored to open new research issues for academia and industries.

- Next G Alliance

At the end of 2020, ATIS (Alliance for Telecommunications Industry Solutions) launched the Next G Alliance in the United States. ATIS aims to promote 6G leadership by putting the basics of 6G in North America [65].

It focuses on technological commercialization, which encompasses research and development, production, standardization, and market readiness. Member organizations’ impact on major mobile communications players might be substantial for future standards. The Next G Alliance will examine industrial innovations and standards strategically. We want to get the worldwide community talking about standards and how to work collaboratively between government and business.

Several major businesses rely on mobile technology for their growth. Aerospace, agriculture, defense, education, healthcare, manufacturing, media, energy, and transportation are just a few of the many sectors that the United States relies on more and more as mobile technology advances. North America must maintain its position as the world leader in mobile technology in these critical industries.

## 4. 6G Security Requirements and Proposed Security Architecture

This section discusses the 6G security requirements and the security architecture.

### 4.1. 6G Security Architecture Requirements

The 6G system security architecture has been designed for openness. Because 6G is intended to be a more open network than 5G, the line between inside and outside the network will become progressively blurred. As a result, current network security measures, such as IPsec and firewalls, will not be powerful enough to protect the network from outside intruders. The 6G security architecture should support the basic security concept of zero trust (ZT) in the mobile communication network to alleviate this issue. ZT is a security paradigm that emphasizes the protection of system resources above everything else. ZT presupposes that an attacker may live within the network and that the network architecture is accessible or untrustworthy from the outside. Such an assessment must be made regularly, and actions must be taken to reduce the risk of internal asset loss. Zero trust architecture (ZTA) is a security architecture that uses the ZT concept and comprises relationships between network entities (NEs), protocol processes, and access rules. Therefore, ZTA should be the foundation of 6G security architecture. Some security requirements can be managed to support secure 6G networks using the ZT concept. In the following lines, we explain the security requirements that must be managed and handled by the security architecture in the 6G networks.

1. Virtualization Security Solution: Virtualization security concerns need the use of a system with a secure virtualization layer, which includes a security technology that identifies concealed harmful software, such as rootkits. In addition, the hypervisor must enable total separation of computing, storage, and the network of different network services using secure protocols such as TLS, SSH, VPN, and so forth. Virtual machine introspection (VMI) is a feature of the hypervisor that examines and identifies security risks by analyzing the vCPU register information, file IO, and communication packets of each virtual machine (VM) to prevent infiltration. When using containerization, the operating system should appropriately set the different containers’ privileges and prevent the mounting of essential system directories and direct access to the host device file container.
2. Automated Management System: To manage vulnerabilities caused by the use, update, and disposal of open sources is the most important thing to do when addressing open source security issues. That is why fast detection of threats necessitates an automated management system that can discover vulnerabilities and apply patches. An additional step is needed to ensure that the patched software is applied quickly and securely using the secure OTA technique. Furthermore, a security governance framework must be established to handle (1) open source vulnerabilities from a long-term view, (2) changes in the developer’s perception, and (3) the deployment of security solutions.
3. Data security using AI: To guarantee that AI systems are safe from AML, they must be transparent about how they safeguard their users and the mobile communication system from AML. Creating AI models in a dependable system is the first step in the process. Additionally, a method such as digital signatures must be used to verify if the AI models running in user equipment (UE), radio access networks (RAN), and the core have been maliciously updated or altered by a hostile assault. When a harmful AI model is found, a system must conduct self-healing or recovery operations. The system should also restrict the data gathering for AI training to trustworthy network parts.
4. Users’ Privacy-preserving: Users’ personal information should be stored and used in accordance with agreed-upon protocols between the service provider, the mobile network operator (MNO), the subscriber, and the MNO in order to ensure their safety. Personal information is kept secure in a trusted execution environment (TEE) and dependable SW by the 6G system, which also reduces or anonymizes the amount of information that is made publicly available when it is used. Authenticity and authorization must be verified before MNO releases personal information. Another option is to utilize homomorphic encryption (HE) when dealing with user information so that the data may be accessed in an encrypted form. AI-based solutions, such as a learning-based privacy-aware offloading scheme, may also be used to preserve the privacy of the user’s location and use patterns.
5. Post-Quantum Cryptography: The 6G system has to get rid of existing asymmetric key encryption techniques since quantum computers will make them insecure. Post-quantum cryptography (PQC) solutions, such as lattice-based cryptography, code-based cryptography, multivariate polynomial cryptography, and hash-based signature, have been the focus of many researchers. As part of its PQC study, the US National Institute of Standards and Technology (NIST) is scheduled to pick the best PQC algorithms between 2022 and 2024. In comparison to Rivest–Shamir–Adleman (RSA), the key length presently under consideration for PQC is projected to be many times larger. PQCs are likely to have a larger computational cost than the current RSA method. As a result, it is essential that PQC be appropriately integrated into the 6G network’s HW/SW performance and service needs.

### 4.2. Proposed Security Architecture of 6G

This section presents a description of 6G’s current research. It also addresses an explanation of new modifications to 6G enabling technologies in the three levels (physical, connection/network layer, and service/application layer).

6G network design will differ significantly from 5G in various ways. First, 6G may accomplish network automation and Network as a Service (NaaS). NaaS enables subscribers to customize networks. Key technologies include intent-based networking, end to end software, cloudization, and deep slicing/function virtualization. Second, the fast adoption of cloud-based networks and open source software for core/RAN network components predicts the “full openness” future of 6G. 6G may be the first entirely AI-enabled cellular system. This vision would transform 5G’s “connected things” into 6G’s “connected intelligence,” with AI eventually controlling most network operations and nodes [66]. According to [67], Deterministic Networking (DetNet) or Time-Sensitive Networking (TSN) may help 6G to achieve ultra-Reliable and Low Latency Communications (uRLLC).

**Figure 3:**
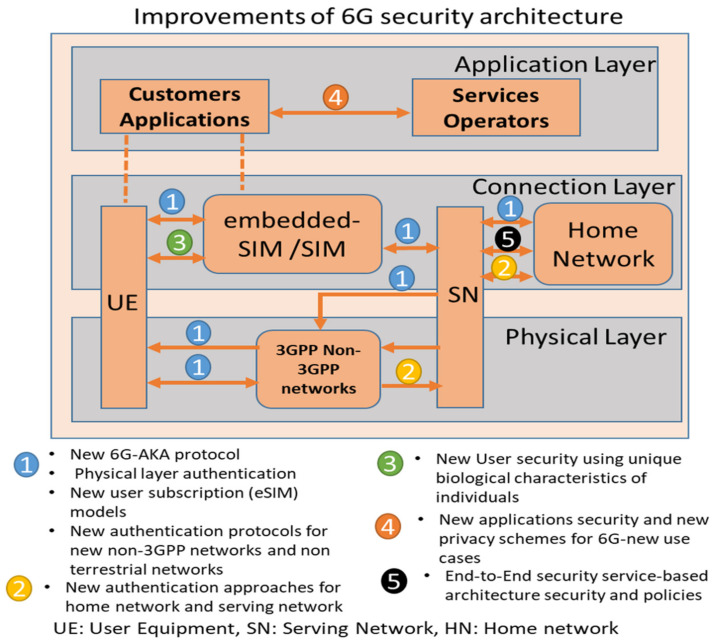
The expected improvements and changes in the 6G security architecture.

6G security architecture will need to adapt to enable new applications and the integration of the space–air–ground–sea network model. The current 3GPP security architecture might need some significant changes, as depicted in Figure 3. Network operators will be critical players to upgrade network access and security architecture. The service providers provide value-added services (online entertainment) and platforms (cloud storage, data analytics) to developers and users. Service providers will upgrade application domain and service-based architecture security. XR/AR game developers will have to increase security for cloud/edge applications or enable new security APIs (following third-party providers’ services). 6G networks may offer mobile storage and other services. Thus, they can help improve service security in many ways. Finally, users may be unaffected by the modifications if they swap devices or register new SIM cards. The security architecture of 6G can be divided into layers to cover all security issues and challenges for all 6G entities. It consists of the physical layer, connection layer, and application layer. Each layer enhances new security functions that can improve the security of the 6G networks. Figure 3 summarizes the improvements of 6G architecture components and layers. It represents the security improvements of the 6G architecture.

We also summarize the security functions and security enhancement of the future 6G architecture as follows:

1. Network Access Security: 6G demands new authentication and cryptography systems. They are 6G-AKA, quantum-safe cryptography, and physical layer security. The motivation for cloud-based and open-programmable networking technologies in 6G necessitates a new authentication so that 6G may use 5G’s security concepts, such as a single authentication platform for open-access networks. Numerous additional functions are required to complete them. For example, a 6G-AKA protocol must guarantee which component, Authentication Server Function (AUSF) or Security Anchor Function (SEAF), would determine authentication in cross-slice communications. 6G-AKA must be able to authenticate an endpoint’s claimed identification in a deep-sliced, programmable networking infrastructure. Physical layer security can defend 6G IoT networks from dangers, including impersonation attacks, and improve network access management. The most significant difference in 6G subscriber administration compared to 5G is introducing a new user identity management approach.
2. Network Domain Security: There will be a need for new open authentication methods because of the extension of 6G to non-terrestrial networks such as satellite and marine communications.
3. User Domain Security: Authentication using biometrics or a password-free service to access control mechanisms has been a long-awaited feature for 6G security. Many applications have relied on password-based security methods for decades. Unfortunately, there are several drawbacks. Some may be easily hacked, expensive to store, and difficult to remember. Brainwave/heartbeat-based authentication might deliver a more secure and improved user experience in the future.
4. Application domain security: Both parties must authenticate themselves for 6G trust networks to operate. Symmetric-key mutual authentication is still in use in 5G. However, 6G networks may benefit from blockchain and Distributed Ledger Technologies (DLT).
5. Service-based architecture security: When it comes to 6G, the service-based security architecture used in 5G is updated to an end to end, service-based, and policy-based security architecture. Domain security is a pillar of the 5G security architecture built on a service-based architecture. Taking this feature to the next level, 6G will use end to end service-based architecture, or perhaps policy-based architecture domain security, to meet the needs of personalization and micro-deployment flexibility while maintaining high levels of security.

## 5. 6G Promising Technologies Security Challenges and Possible Attacks

Some significant technologies have already been proven to be efficient in important essential sectors of the 6G networks. They provide high security, low latency reliability, and efficient communication services to 6G networks. However, most new 6G technologies have higher security and privacy risks. This section analyses the leading technologies in 6G, and the security and privacy requirements for these technologies [68,69].

### 5.1. 6G Physical Layer Technologies

The proposed methods for securing the physical layer depend on the random physical characteristics and the noise surrounding wireless networks. However, the flexibility of PLS mechanisms, particularly in resource-constrained conditions, with the possibilities of disruptive 6G technologies, may pave the way for a new era of PLS in the 6G era [70].

- Terahertz communications (THz)

The THz ranges between 0.1–10 THz. These frequency bands combine optical waves with a vast spectrum and microwave that can support high transmission rates, robust anti-interference, and simple integration of sensing and communications. THz communications are used initially to fulfill system needs for transmission speeds in the order of Tbps. THz communications will be a valuable continuation of existing transmission methods. They will be essentially used to communicate with latent holographic communications, small-scale communications, ultra-high-capacity data, and short-range transmission with ultra-high-speed are only a few of the application opportunities. Positioning with high accuracy and sensing with excellent resolution using THz communication signals are other demanding applications. Many significant technologies and difficulties for THz communication are listed below. There are three typical transceiver architectural designs: direct modulation architecture, solid-state frequency mixing modulation architecture, and optoelectronics modulation architecture. The main design concerns for architecture are excellent compatibility, excellent energy efficiency, and cost-effectiveness. In terms of RF-end components, a THz system’s primary elements include a THz signal source, a mixer, a multiplier, a detector, and an amplifier [71].

The advantages of THz spectrum use are highlighted by Huang et al. [72] as follows:

1. Firstly, the THz communication technology may support 100 Gbps or greater data.
2. Secondly, eavesdropping would be decreased, resulting in greater communication security due to the narrow beam and short pulse length of the transmitters.
3. Thirdly, it is constrained to attenuate THz vibrations by specific materials.

At the moment, THz operating frequencies and output power do not satisfy commercial criteria for high system efficiency, low energy consumption, and extended service life. Advanced semiconductor materials such as silicon germanide (SiGe) and indium phosphide (InP) must be investigated. Furthermore, THz systems need real-time Tbps transmission rates in baseband signal processing. Therefore, developing high-speed processing technologies for baseband signals is simple and consumes little power. In terms of antennas, most high-gain antennas today have massive reflectors, pushing the development of downsized and arrayed THz ultra-large-scale antenna technology [73]. However, the mm-wave radio bands are broadly utilized in 5G networks. The requirement for very high transmission rates in a 6G environment makes such bands sufficient. In this regard, the RF bands are practically completed and cannot be used for future technologies [74,75]. The 0.1–10 THz range is used by Terahertz technology, with the available spectrum more utilized than the mm-wave spectrum. It also uses electromagnetic waves as well as light waves.

The authors in [76] confirmed that an unauthorized user could capture communications by strategically placing objects in the transmission path, scattering the radiation toward the user. In that article, it is suggested to distinguish the channel’s backscatter to identify certain eavesdroppers but not all. Additionally, [77] suggested that the authors investigated the THz propagation multipath nature to improve security. The authors demonstrated that by dividing data transmission over several routes, the probability of message eavesdropping might be mainly decreased, even when many eavesdroppers cooperate, at the risk of a slight reduction in connection capacity. This method may be investigated for the transmission of private data or securing the key exchange process in THz networks.

Additionally, [78]’s researches managed authentication at the physical layer in vivo nanonetworks operating at THz frequencies, using a distance-dependent-path loss-based authentication technique. The authors confirmed how path loss might be utilized as a fingerprint with a THz time-domain spectroscopic setup. Overall, new physical layer solutions used the THz frequencies’ electromagnetic signatures to perform physical layer authentication. These solutions would benefit THz wireless and incorporate new countermeasures in transceiver designs.

Strianti et al. [79] indicated that the THz communication power consumption was considered a significant challenge. Furthermore, 6G cells must be sized from small to tiny to accommodate the new technology requirements, which means that complicated hardware and architectures must be built [80]. THz also had its privacy and security problems as with other technologies. THz’s security and privacy problems were mainly focused on authorization and different types of abnormal behaviors. In [81], Akyildiz et al. discussed ideas such as electromagnetic signatures for various THz frequency bands employed in the physical layer authentication procedures. However, THz was assumed to be difficult to manage eavesdropping and attacks. In [82], Ma et al. claimed that eavesdroppers might capture THz signals using narrow beams. They introduced some resisting solutions against these narrow beam attacks.

- Visible light communications (VLC)

VLC is a practical technology that can meet the 6G wireless network requirements [83]. In addition, VLC has been investigated in many fields for a long time, such as in indoor localization solutions and Vehicle-Ad -Hoc-Network (VANET) [84]. The VLC technology has wide bandwidths that make it tolerate the interferences compared with the RF with severe interference and notable latency [85]. VLC security standards follow basic security requirements for all wireless networks. They inquire about securing VLC communications from eavesdropping, DoS or jamming, and node compromise attacks. Confidentiality, Integrity, Authenticity, Availability (CIAA) criteria are described as [86]:

1. Confidentiality: It restricts the access to data only for intended recipients and prevents the information from being disclosed to side organizations.
2. Integrity: To ensure the correctness of the information sent while the authenticity verifies the network node identification.
3. Authentication: Depends on identity authentication and information authentication. The first one is to ensure the identity of the access person, while information authenticity provides that no one changes the transmitted information. Both authentication parts are required to ensure the security of the information and resources.
4. Availability: Is the ability of users to connect to the wireless network at any time and from any location.

**Figure 4:**
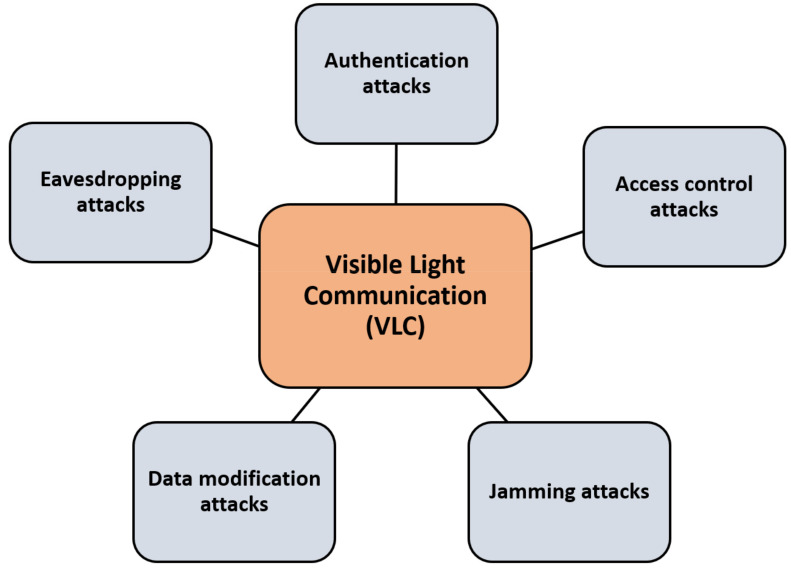
The 6G visible light communication technology attacks and threats.

Moreover, signal overlap may result in diverse transmitter signals overlapping; therefore, authenticity, integrity, and accessibility may be at threat. The physical characteristics of the light communication medium are principally affected by the two lowest levels (PHY layer and MAC layer). The attacks in this technology target the physical layer by eavesdropping, jamming, and capturing the transmitted data. Other control access attacks happen due to authorized access for the wireless medium, with authentication attacks. Figure 4 shows the most common attacks in 6G visible light communication technologies.

In [87], Chen et al. proposed a LiFi VLC solution, allowing concurrent access to many simultaneous mobile users and delivering very high-speed and cost-effective services. However, many weaknesses that are limiting VLC technology advancement still exist. We propose these shortcomings: Indoors applications, for example, should be the primary use case for VLC since excessive natural light would affect transmissions. VLC-related privacy and security problems involving malicious activities and communication are critical issues.

Due to the vulnerability of VLC schemes to sniffing and eavesdropping attacks, network confidentiality is at risk [88]. Additionally, VLC techniques present prominent features from RF systems that should be considered while designing PLS mechanisms. VLC channels, for example, are natural channels, and the VLC based systems require high power restriction on unbounded inputs. In conclusion, these limitations should be solved to evaluate the network performance and optimize PLS techniques in VLC systems. Additionally, research performed in [89] found that VLC systems are more susceptible in areas with high reflections.

In [90], the authors demonstrated how linear precoding might enhance the secrecy performance of a VLC Multiple-Input Multiple-Output (MIMO) system in terms of the available rate of secrecy. However, the transmitted signal’s peak-power limitation was addressed, and only discrete input signaling methods were utilized. Additionally, they explored a blind PLS watermark method in which green, red, and blue LEDs and three tuned color photodiodes were used to increase the VLC system secrecy by including a receiver that works by jamming the spread spectrum watermarking approach.

In [91], Pathak et al. highlighted that the victim’s line of sight should be on the current VLC process if the adversary proposes to conduct an attack. This would simplify the problem for attackers. Ucar et al. [92] introduced a SecVLC protocol to protect the privacy of data transmissions over vehicular networks. The precoding technology for VLC connections was proposed by Mostafa et al. [93] to guarantee the physical layer communication efficiency. Figure 4 shows the 6G visible light communication technology attacks and threats.

- Molecular communication

**Figure 5:**
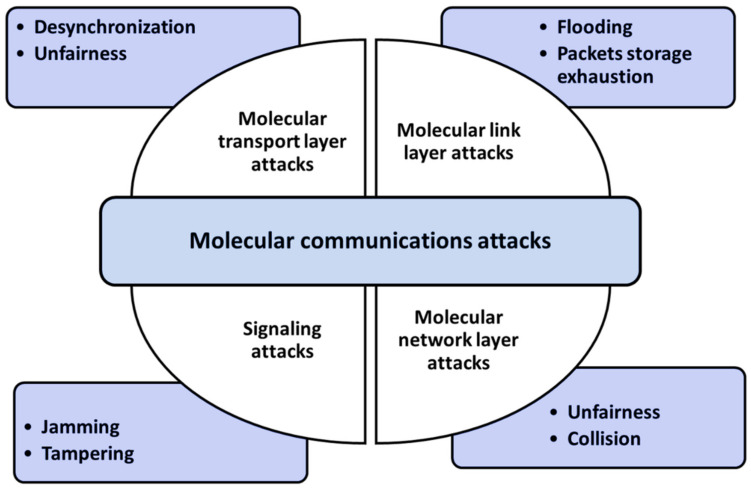
The 6G molecular communication attacks and threats.

The technique of molecular communication is a promising 6G technology. However, the method has not matured yet and is still in its beginning phases. The fundamental principle of molecular communication technology is to transfer information using biological signals. Nakano et al. [94] proposed a technique of mobile-molecular communication that allowed the sender, recipient, and nodes to cooperate during movement. Several privacy and security problems have been identified concerning communications and authentication processes in molecular communication. Different attacks target molecular communication security by dropping the transmitted data or altering the information between sender and receiver. These attacks are classified into four categories [94]: transport layer attacks, link layer attacks, network layer attacks, and signaling attacks in the physical layer. The molecular transport layer is responsible for session management and security. We may imagine various security concerns, such as desynchronization and flooding, that are equally frequent in traditional communication networks. Collision and unfairness are two common link layer threats. Collision attacks are addressed at the link layer using error–correction methods. On the other hand, attackers may execute unfairness and collision attacks when the diffusion method sends information. The concept of the molecular network layer identifies the entities involved and specifies the essential capabilities. These functionalities are responsible for the formation and routing of molecular networks. For example, molecular packet loss handling attacks induce packet loss due to a lack of molecular packet storage. This type of attack is known and constitutes a security concern. Some attacks, such as tampering and jamming, can be generated depending on the input and output associated with the bio-nano device statues. The most common solutions for defending against these attacks are frequency hopping methods and spread spectrum. However, these solutions cannot be applied to molecular communication; molecular communication requires ad hoc solutions to defend bio-nano devices against attacks [95]. Figure 5 summarizes the molecular communication of different attacks at different layers.

Farsad et al. [96] claimed that an adversary might disturb this kind of transmission medium, and only a few researchers have studied the security of molecular communication. In [97], Lu et al. introduced a novel coding and encryption system to improve and enhance network security and privacy. Moreover, Loscri et al. [98] presented several possibly practical molecular communication guidelines that would help create novel security methods to ensure data privacy and authentication. They explored many ways for attacking the molecular medium at various levels, such as desynchronization, jamming, and flood attacks. However, the advances in molecular networking technologies for the 6G networks need more effort. This technology was predicted to do what conventional communication cannot do.

### 5.2. AI/ML Technology

Recently, AI and ML have been marked as necessary components of the network architecture of all 6G networks technologies. As a result, artificial intelligence received much attention in the 6G networking. AI/ML in the 5G networks is implemented in locations with vast training data and efficient computing cores. However, AI/ML has become a significant entity of the 6G networks. AI and ML are used to secure various frames of 6G’s security defense and protection. The use of AI and ML in security makes the security solutions more autonomous and more accurate with predictive capabilities for security analytics. This sub-section addresses some of the challenges associated with AI/ML in the 6G system [99].

1. Trustworthiness: The reliability of machine learning models and components becomes important when AI handles network security.
2. Visibility: Monitoring security functions based on AI and ML in real time to ensure control and credibility.
3. Ethical and Legal Aspects: Optimization techniques based on AI can limit some customers or applications. AI-powered security solutions are uniform in their protection of all users or not; who is responsible for security services’ failure controlled by AI.
4. Extensibility and viability: Secure data transfers are necessary to ensure the privacy of federated learners. Scalability of the required computing, communication, and storage resources is a challenge for AI/ML.
5. Controlled security tasks: Much overhead may result when AI/ML security solutions are associated with significant data processes.
6. Models’ flexibility should be secure and flexible in the learning and inference steps.

**Figure 6:**
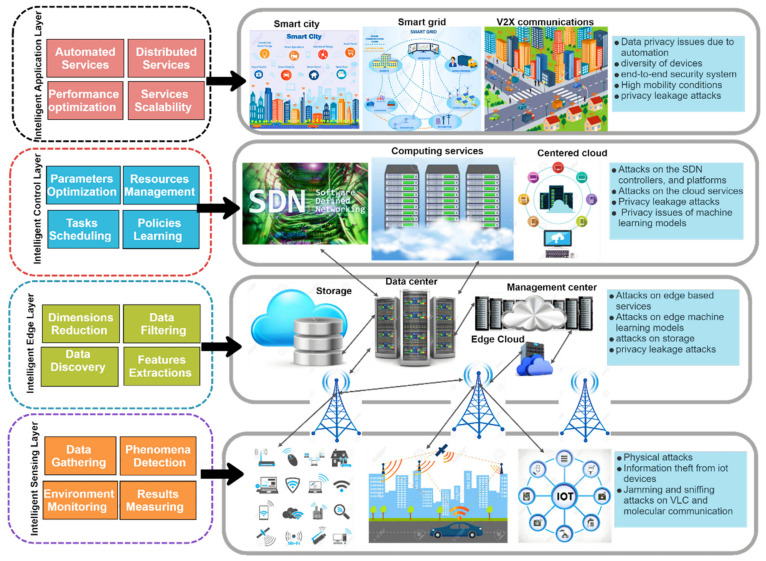
The 6G AI/ML security architecture, and different attacks in each layer.

The expected intelligent 6G system is for evolved AI mechanisms and techniques to support high service requirements, needed capabilities, and new use cases’ requirements. The 6G secured architecture-based AI/ML is shown in Figure 6 and summarized as follows [100]:

- Intelligent sensing layer (Radio layer)

This layer collects information from the physical world. Many connected devices collaborate to share information through wireless media to monitor physical phenomena. The collected data are then passed to the higher layer for further processing. This layer introduces some attacks on the small connected IoE devices. These attacks are physical, theft information from devices, attacks on visible light communications, and sniffing attacks.

Advances in circuits, antennas, meta-material-based architectures, and the rapid development of AI chips have uncovered a paradigm change in the hardware design of 6G transceivers, allowing hardware to be decoupled from transceivers’ algorithms. As a result, the transceiver algorithms may dynamically configure and update themselves in response to the changes in the environment and hardware. Intelligent radio will use cutting-edge AI/ML approaches to solve difficulties in the wireless domain, such as accurate channel modeling, agile physical layer design, dynamic spectrum access, sophisticated network deployment, optimization, and autonomous orchestration. Thus, suspicious activity by malicious nodes must be foreseen during secure radio communication procedures [101].

- Intelligent edge layer

The edge layer extracts a feature from the collected data, classifies it, and analyzes it. The edge layer attacks target the machine learning models and edge services. Moreover, some security-related issues are connected to privacy and data storage. Edge intelligence (EI) applies AI/ML algorithms to gather, store, or analyze data at the network edge [102]. There are several advantages for using an edge server in EI, such as quicker feedback, decreased latency, and cheaper costs since it gathers data produced by several connected devices and shares it with other edge servers for training models. Since the results of AI/ML algorithms are heavily data-dependent, EI is particularly vulnerable to various security vulnerabilities. A variety of assaults, such as data poisoning or evasion, or privacy infractions, might take advantage of this reliance, compromising the AI/ML applications’ outputs and undercutting the advantages of EI.

- Intelligent control layer

This layer controls the tasks scheduling, resources managements, parameters optimization, and policy learning that results in various attacks. The control layer attacks target the SDN, cloud computing services, and centered cloud services. Moreover, the SDN attacks on the SDN controllers, interfaces, and machine learning attacks on the intelligent learning models are considered critical security issues in the upcoming 6G networks.

- Intelligent application layer

Automated and distributed services in the application layer introduce several attacks. Due to automation, data privacy issues are related to smart cities and vehicular communications. The diversity of devices incorporated in the application layer requires a high-security level [103]. Since 6G has such a wide variety of needs and is expected to fully automate network and service management from end to end (E2E) using AI, a significant shift in network service orchestration and management is required in 6G design [104,105]. Intelligence network management may be achieved using the ETSI ZSM (zero-touch network and Service Management) architecture for 5G [106]. These intelligence network management deployments have several security challenges. DoS and Man-in-the-Middle (MITM) attacks may all be introduced using closed-loop network automation. DoS attacks may be carried out by progressively increasing the capacity of virtual machines by faking excessive demand on virtual network functions (VNFs) (VMs). Fake fault events and intercepting domain control messages may divert traffic via malicious devices in an MITM attack. The sent data may be tampered with to carry out deception attacks. As a second example, if 6G networks employ intent-based interfaces such as ZSM, which might be sensitive to information disclosure, unwanted configuration, and abnormal behavior, assaults can occur. As a result, unauthorized access to system security goals (e.g., privacy, confidentiality) might lead to additional assaults and compromises. Changing the mapping between intent and action or lowering the security level in intent-based interfaces may put the whole management system at risk. Similarly, a misguided goal could have the same effect.

**Figure 7:**
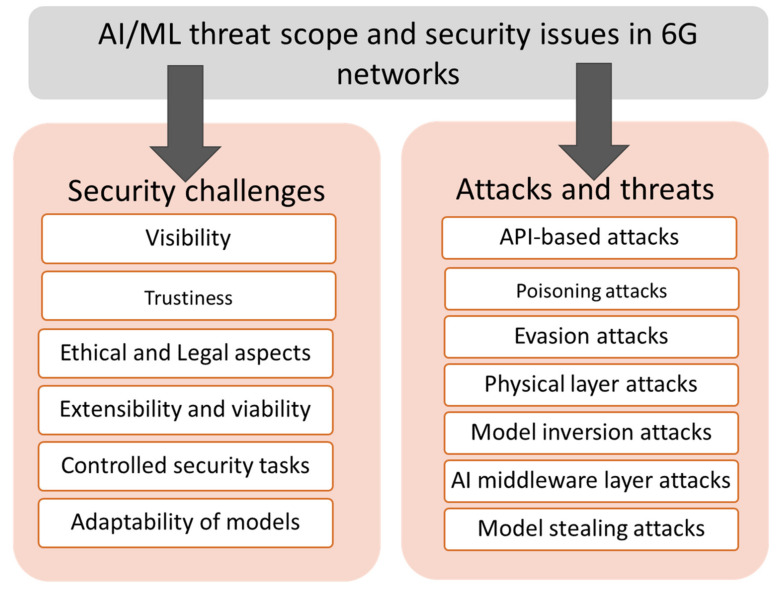
The 6G AI/ML security challenges and threat scope.

However, more complicated attacks have been developed in recent years, such as those against federated learning. 6G networks depend significantly on AI and machine learning technology. However, AI and machine learning will initiate AI/ML-related threats. These attacks are directed at both the training and test phases. Figure 7 summarizes the security challenges and known AI/ML attacks and threats in 6G networks based on AI and ML. During poisoning attacks on the AI training stage, the attacker may handle the trained data by inserting specifically designed incorrect samples, thus affecting the result of the learning method. Such injections of prepared samples may create intelligence services security to exceed resource needs and misclassify services. Evasion attacks bypass the learned model during the test stages by injecting the tested data. Finally, the model inversion attacks attempt to obtain training data from the targeted machine learning models.

In contrast, model extraction attacks use model parameters to reproduce comparable models. Finally, infrastructure and physical attacks aim for data tampering, malicious interruptions, and inefficiencies in the communication and computing infrastructure. These attacks are initiated to trouble decisions and data processing and bring the AI systems down. Significant exposure to AI frameworks exploits flaws or conventional attacks intended against their firmware, software, and hardware components. For example, API threats involve an adversary querying a machine learning model to obtain forecasts on the input vectors of features. In addition, other AI attacks include recovering training data from a model, revealing model architecture to expose the model confidentiality, and using the model output to predict the training data [107].

There are many methods available to prevent AI/ML risks. Adversarial training augments resilience by introducing disturbed instances resembling threats into training data. Another protective approach is defensive distillation, which depends on information transfer across neural networks using software labels considering the previously trained output of a network and indicating the different classes. These software labels are also used for training rather than as complex labels to assign all data to a single category. Both of these methods are successful against both evasion and hostile attacks.

On the other hand, security protection against poisoning during the training phase is risky to safeguard data integrity and authenticate the data’s origin. Blockchain technology offers a distributed, transparent, and secure platform for data exchange. Likewise, shifting target defense and input validation are used. Additionally, the latter is advantageous against hostile assaults. Furthermore, the security solution to avoid inversion attacks effectively restricts information to algorithms via machine learning APIs [108]. Zhang et al. [109] introduced various AI-based techniques used in multiple physical levels, including artificial neural networks, Kmeans, and uncontrolled learning. By optimizing interoperability, these methods could enhance the effectiveness of physical layers and increase the field of prediction and safety. Sattiraju et al. [110] introduced an unsupervised machine learning solution to improve the authentication process and the physical layer security. Hong et al. [111] introduced a novel design for the antenna that could improve the physical layer communication classification tasks to prevent data leakage.

Furthermore, Nawaz et al. [112] pointed out that the protection of 6G links is enabled through encryption and machine learning schemes. Zhou et al. [113] also explored AI technologies; they claimed to detect threats in advanced computing in greater detail. However, they recommend additional exploration.

Dang et al. [114] claimed that AI might support in identifying network problems in the 6G security and provided prevention approaches and protection solutions. Tomkos et al. [115] noted that network edge devices could exchange information to increase network security using federated distributed AI in 6G networks. Zhang et al. and Zhu et al. [116] highlighted that there could be increased privacy breaches because of the impact of data exchanges on some machine learning techniques.

### 5.3. Quantum Communication

Another technology for a communication system with several enormous applications in 6G networks is quantum communications. Security and reliability are considered two significant interests for quantum communication that can be improved vastly. The quantum status will be changed if an attacker changes or duplicates something in the quantum communication. Theoretically, quantum communication supports perfect reliability and is highly appropriate for long-distance communication with correct innovation. It gives various innovative solutions that enhance communications to a standard level. The adversaries have quantum abilities inside the threat environment of quantum-based attacks [117,118].

Integrating post-quantum crypto solutions resistant to quantum attacks into IoT devices is always a challenge. As a result, devices based on quantum cryptography pose a problem in the future 6G post-quantum standards. In classical information sharing, Oblivious Transfer (OT) enables a sender to transmit one of the possible information pieces to a recipient while staying unaware of which data was sent. Quantum information cannot maintain this feature since any leakage would destroy the connection.

In quantum cloning attacks [118], the attackers take a random information state to create an identical duplicate without changing the information’s original condition. Although perfect quantum state copies are forbidden, it demonstrates that a quantum state may be duplicated using different excellent cloning methods with the most excellent precision. Quantum cloning attacks may occur in high-dimensional quantum key distribution systems as a kind of quantum hacking in a secure quantum channel.

Quantum communication still does not provide a solution for all privacy and security matters. Although notable advances have been achieved in quantum encryption for quantum communication, operation mistakes and fiber attenuation are complicated challenges. Hu et al. [119] assumed the need for several quantum cryptography methods and other technologies to ensure a reasonable level of quantum communication security. These technologies are similar to the key management process, secret key sharing, and direct quantum communication security. The security of direct quantum communication is provided in Zhang et al. [120]. They allowed the encrypted message transmission through a direct channel without sharing the secret key. Nawaz et al. [112] proposed a novel quantum mechanism that uses the quantum key distribution to ensure key security.

### 5.4. Distributed Ledger Technology

The expected cooperation between DLT and 6G may implicitly impact the safety flaws in blockchain and smart contracts in 6G networks. These attacks occur due to software development errors, language restrictions, and network connection security flaws [121,122,123,124].

**Figure 8:**
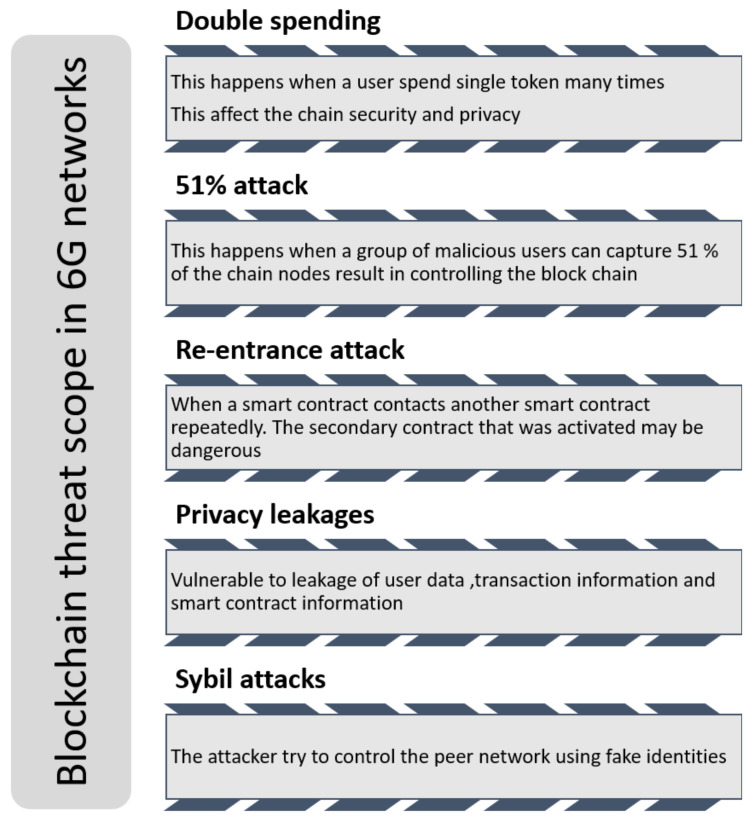
The 6G blockchain technology attacks and threats.

Moreover, both public and private blockchain systems may be changed by similar attacks. As a result, they lead to reduced accuracy, financial losses in Bitcoin, and more severe system availability. The following are the most significant security breaches on blockchain and intelligent contract platforms, as shown in Figure 8.

1. Attack of majority: This is called a 51% attack; when malicious people take 51 percent or more of blockchain nodes, they may succeed in network control. By majority attack, attackers may modify the transaction history and block the confirmation of future official transactions. Therefore, the majority voting blockchain systems based on consensus are generally vulnerable to 51% attacks [125].
2. Double-spending attack: A key component of most blockchain systems is spending the cryptographic token. However, since there are no physical notes, there is a threat that a user spends a single ticket several times. These are recognized as double-spending attacks, and systems based on the blockchain should provide solutions to prevent them [125].
3. A re-entrance attack: This happens when a smart contract contacts another smart contract frequently. The secondary smart contract that was initiated may be vulnerable. Such an attack, for example, was conducted against the Decentralized Autonomous Organization (DAO) in 2016. Unknown hackers stole USD 50 million in Ethers [125].
4. Sybil attacks: This type of attack happens when attackers or many attackers try to capture a peer-to-peer blockchain network by establishing fake identifications. Sybil attacks are more common in blockchain systems with restricted and automated member addition methods [125].
5. Privacy attacks: Smart contracts and blockchains are prone to security and privacy concerns, including transaction data leakage, smart contract logic leakage, user privacy leakage, and privacy leakage during smart contract execution.

Specific blockchain nodes may impose stringent privacy rules and promote excessive openness, exposing the pricing information and leakage of sensitive secrets. Furthermore, the business logic of the company must integrate with blockchain. For example, bonuses and commission attacks can happen when the company information is saved in smart contracts and shared with the competitors. Based on the earlier mentioned attacks, smart contracts and blockchains are vulnerable to various additional security threats. These threats include exception disorder, destroyable contracts, call stack attacks, underflow errors, insufficient randomness, broken authentication, overflow errors, security misconfiguration, broken access control, and unbounded computational power. Blockchain technology has broad applicability in a 6G network; for example, distributed technology for a ledger, decentralized network, and spectrum sharing [126].

In [114], Dang et al. emphasized that the decentralized network based on blockchain technology facilitated network administration and increased performance. In [127,128], the author recommends using blockchain in the distributed ledger to enhance and improve network security and authentication. It could be one of the critical technologies that mainly disturb Internet users [129]. Implementing the blockchain in a shared spectrum system might solve the difficulties of spectrum monopoly and the low-spectrum utilization, thus ensuring optimized spectrum usage while solving the network security and privacy problems.

The novel architecture of the Mobile-Service-Authorization based on blockchain was presented by Ling et al. [130]. This architecture enhances the radio access network architecture to ensure network safety and efficiently manage network access among different network entities. Kotobi et al. [131] suggested enhancing the cognitive radio safety and media access protocol by utilizing the blockchain to access the free licensed radio spectrum. However, the decentralization of the 6G network architecture was achieved. Therefore, the attackers possibly changed the records if more than a defined percentage of nodes represents 51% of the total nodes controlled by the hacker. Then, security flaws occurred. Moreover, security flaws could happen when a trusted third party is not included in protecting the network storage and data monitoring [132]. Ferraro et al. [133] indicated that it might significantly affect blockchain security due to the hash chain capacity to validate transactions across blockchain networks. Table 2 summarizes the 6G technologies, security challenges, and the fundamental contributions.

## 6. 6G Applications’ Security Challenges

**Figure 9:**
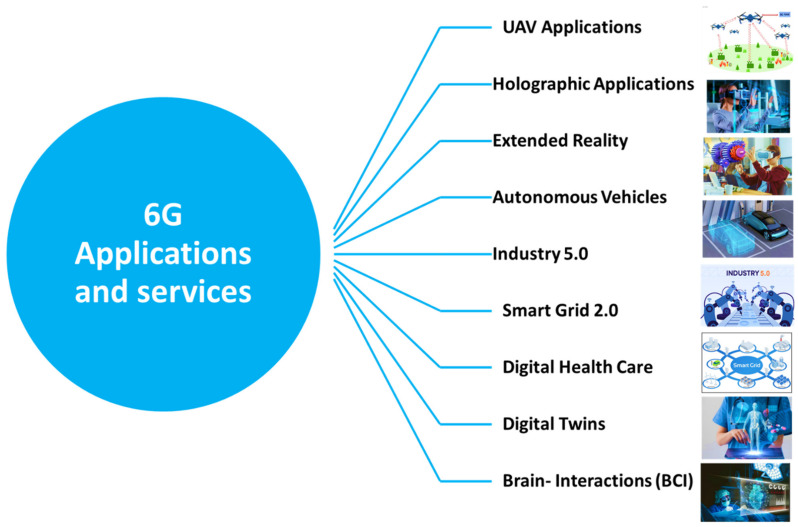
The most essential 6G applications in different technologies.

Due to the high communication requirements and needs of the 6G applications, many applications and services have very demanding performance and extraordinarily stringent security requirements. The interaction between general performance expectations and security needs to become increasingly more complex as highly competent, ubiquitous attackers and malicious activity become more prevalent. The following subsections discuss the most essential 6G applications, as summarized in Figure 9. Moreover, they present the future 6G advances and challenges for different 6G applications [134].

### 6.1. Unmanned Aerial Vehicle (UAV) Applications

Though an autonomous drone system has not yet been completely implemented due to the constraints of 5G networks, 6G networks might realize the full capabilities of those systems. Unfortunately, some cyberattacks on these systems also occur. This sub-section investigates the UAV challenges and requirements within 6G communications to support high secured systems. UAV networks are different from other 6G applications where UAV nature is unmanageable and highly dynamic. UAV features and requirements are highlighted as follows [135,136]:

(1) High altitude: UAV systems always fly higher than typical mobile users and base stations. There are no obstacles in the wireless connection between the base station and the UAV. Thus, air–ground channels are less susceptible to scattering and have lower route losses than the traditional terrestrial channels. The Line of Sight (LoS) channels provide more excellent dependability and lower route loss in air–ground transmissions than non-Line of Sight (NLoS) terrestrial communications. However, LoS channels cause significant interference with other nodes coexisting in the wireless network. Hence, the three-dimensional location in the space for UAVs must be studied to take advantage of the LoS channels.
(2) High mobility: Typically, nodes in traditional communications are located in fixed places. UAVs are controlled to fly at high speeds in three-dimensional space remotely. UAVs can be deployed in diverse ways to create wireless connections. This feature is more worthwhile for emergency cases such as military activity and disaster relief. Moreover, the mobility of UAVs may be used to maneuver closer to the targeted user to maximize the gain of the channel and avoid obstructions. Thus, the UAV’s trajectory may be optimized for improved communication performance.
(3) Limited Energy: UAVs have limited energy due to their weight and size limitations. Additionally, UAVs must supply energy for both communications and push simultaneously. Thus, the propulsion energy consumption required to keep the UAV flying is much more than the conventional energy consumption. Consequently, it requires an energy-efficient design to maximize its lifetime.

Li et al. [137] discussed that the SDN controlling systems could control UAV networks. Hooper et al. [138] mentioned WiFi attacks, which an adversary of Tiro may exploit. Fotouhi et al. [139] indicated how autonomous drone systems arise, such as attacks through spoofing, eavesdropping, DoS, and hijacking attacks. Therefore, different measures are required for improving security.

Since 5G, UAVs have become popular in many applications. UAV technologies are being employed with AI and 6G, and many innovative use cases, including passenger taxis, automated logistics, and military operations, are growing. The restricted resources availability, such as computing and latency-sensitive applications in UAVs, and lightweight safety measures should be used to meet the low latency demands. Issues such as high scalability, device variety, and high mobility need to be considered while designing UAV security.

6G enables UAV functionalities based on Edge AI and AI, such as collisions avoidance and trajectory planning, optimization of routes, and swarm management. Therefore, it is essential to use mechanisms to prevent threats associated with AI. Due to the unmanaged UAVs’ nature, they are very susceptible to different physical attacks. For example, an opponent may capture the UAV physically via interference control signals or physical devices, then take critical data from inside the UAVs. In addition, UAVs will support sophisticated communication capabilities concerning other intelligent devices. For example, drones may be employed to conduct coordinated attacks. Such attacks may vary from cyberattacks to physical attacks.

### 6.2. Holographic Applications

Holographic communications will be widely employed in various industries, including entertainment, healthcare, education, and manufacturing. Wireless networks must handle massive throughput due to multidimensional interactions involving hundreds of different data streams, all running simultaneously in holographic applications. When data are lost during holographic targeted cure or remote microsurgical operations, it is necessary to retransmit the information. Network communication security and reliability should be significantly enhanced [140].

With holographic telepresence, users can see distant people and objects in real time in three dimensions (3D) with a degree of realism equal to or greater than their actual presence. Holographic communication is only possible with extremely high bandwidth. The demand for bandwidth will rise in tandem with the growth in holographic communication devices. Because of this, security mechanisms used for holographic transmission should not add to the already-exhausted bandwidths. While developing holographic communication security measures, reduced operational expenses and device variety must be considered. In the context of holographic telepresence, maintaining privacy is still a critical concern [141]. When a holographic image is projected on a distant site, the required level of confidentiality must be taken into account. The remote presenter must provide additional privacy protection solutions so that customers can secure their privacy.

### 6.3. Extended Reality

Extended Reality (XR) is a combination of virtual and realistic settings covering Virtual Reality (VR), Augmented Reality (AR), and Mixed Reality (MR). 6G supports XR’s progress by offering to utilize them in various applications, including online gaming, virtual tourism, online education, entertainment, robot control, and health care. The management of personal information is an essential security element of XR that will contain credit card numbers or acquisitions and more sensitive information such as emotions, behavior, judgments, and physical appearance. Therefore, the necessary degree of data accountability becomes a fundamental need for 6G networks to gather, store, secure, and share personal data [142].

When XR security measures are deployed, high network scalability, minimal overhead, and device diversity must be considered. The degree of security measures in the XR program may vary depending on the application. For example, military services need the most significant security level (i.e., data encryption, robustness, multifactor authentication, user access restriction), whereas entertainment apps require less security. False experiences are another vital security problem linked particularly to XR. If incorrect or fabricated data are utilized in XR apps, the entire XR experience will be unsuccessful.

5G networks have enhanced AR and VR experience by increasing bandwidth and lowering latency. Unfortunately, releasing VRs on 5G networks still has various challenges within the 6G network. For example, the VR/AR cloud scan currently provides users with innovative features, but the delay is a big issue, and the associated uncertainty leads to further difficulties. Deployment of VR/AR through the cloud provides more user-friendly and accessible services, but 5G bandwidths make it reasonable to compress pictures. Therefore, we must move to the 6G networks to send massive uncompressed photos or movies for real-time transmission. The 6G networks further enhance VR and AR experience. Sensor networks are utilized to gather sensed information and to give users feedback. XR represents extended reality, which refers to any real-life and virtual integrated environments and interactions between people and machines created by computer systems and devices. The XR on 6G technology is expected to be combined with the enhanced Mobile Broadband (eMBB) and URLLC communications, which might be known as the Mobile Broad Bandwidth and Low Latency (MBBLL). URLLC and eMBB provide remarkable privacy and security problems in multi-sensory XR systems, including harmful behaviors.

Chen et al. [143] claimed that the reliability of a network with ultra-low latency was needed to address network dynamics. Chen et al. also observed that some cyberattacks were still too complex to protect against. Thus, sensitive and confidential data could still be disclosed. Furthermore, Hamamreh et al. [144] enhanced a method for improving security against this URLLC attack. Moreover, Al-Eryani et al. [145] developed the innovative multi-access approach DOMA, capable of being applied for multi-sensory XR solutions to extend the capability for excellent access to 6G XR devices. However, authors in [146] emphasized that more significant consideration should be given to the privacy, security, and secrecy of eMBB. Yamakami et al. [147] proposed a 3D system modeled for the risks posed to privacy in many XR systems. Pilz et al. [148] indicated that multi-sensory XR systems could manage connected services to preserve confidentiality and security.

### 6.4. Connected Autonomous Vehicles

6G networks will be vast, offer the best experience, and be applicable in a wide range of scenarios, allowing connectivity to be available anywhere. The architecture of the 6G network, with an emphasis on access and core networks, should be our primary concern. The access network design must be reduced and made sufficiently elastic to provide the essential capabilities to minimize processing latency. In addition, the research might concentrate on intelligent control mechanisms driven by requirements and radio resource management, showing the necessity of a software-based, service-oriented approach to design. Architectures for distributed, decentralized, and autonomous networks can create universal, adaptable networking methods for the core network. Many essential technologies are included in the distributed autonomous network architecture, including user-centric control and management, deep edge nodes, and networking. Decoupling between networks and services is also possible because of a lightweight access network architecture driven by requirements, an intelligent control mechanism, and radio resource management. As a result, new technical concepts such as digital twins in networks must be promoted. To improve the automation of the network, however, traditional network optimization and innovation must have a significant impact on network operation. Still, they come at a high time cost since they must be deployed in live networks. Digital twins can help network development improve visibility, more accurate modeling, prediction, and more intelligent control. It is possible to engage and map digital twin networks in real time since they combine physical and virtual network components. The twin network uses closed-loop simulation and optimization to manage the physical network, and the issue here is to make good use of network data and model networks. Because changes in network architecture will have a notable impact, it is equally important to incorporate new technical elements and integrate them into existing networks. The second use is the connected autonomous and robotic systems on the 6G network. Self-driving is a significant 5G network application. However, automatic driving alone is not enough on 6G networks; it requires a robust self-response system. In addition, it should integrate intelligence throughout the network and include AI logic in the network design, allowing us to control and connect all internal components using AI dynamically. Industry 4.0 was discussed by Jamwal et al. [149] to minimize human intervention via using automatic control systems in industrial applications. Recently, a plant has been created to autonomously manage a whole system’s communication, calculations, storage, and resources control. In this case, mobile actuators, cloud services, and databases make it a fully independent system that can be included in the automated factory. The privacy and security challenges of these two applications are discussed in the next section.

Challita et al. [150] provided secured real-time operations on autonomous drone systems by proposing a network-based artificial neural system. Furthermore, Sanjab et al. [151] offered a new mathematical model that could assess autonomous drone systems’ trustworthiness and upgrade them. In contrast, Sun et al. [152] introduced a novel way of communication that might avoid eavesdropping attempts. Finally, Kim et al. [153] proposed a framework that would protect the privacy of the UAV network for managing the problems of authorization and authentication.

In autonomous driving applications, security and data protection problems include different elements such as security and privacy challenges at the system level, the privacy of the location, and vulnerable system consumption. Xu et al. [154] introduced an Efficient and Privacy-Preservation Truth Discovery (EPTD) technique for vehicle applications to protect user security and confidentiality. Ni et al. [155] presented a two-factor authentication approach for autonomous vehicles to eliminate security breaches and reduce the vehicle’s theft threat. Ding et al. [156] developed a new fuel-efficient planned path that might resolve concerns with power consumption in automated driving applications. In [157], Wang et al. highlighted that intruders might target autonomous vehicles by employing brute force attacks and packet capturing attacks. Furthermore, Tang et al. [106] surveyed many machine learning approaches proposed for autonomous vehicles.

Almost 50 major car technologies have spent significantly on autonomous driving technologies. Soon, the world will experience independent, dependable, safe, and economically successful driverless vehicles. A new service ecosystem is being created by introducing Connected Autonomous Vehicles (CAV) technology, such as driverless taxis and driverless public transport. The complex CAV security problems may be classified in three areas: at the vehicle level, the supplier chain, and data collection. First, attacks at the vehicle level may occur via the capture of car sensors, physical controls, and V2X communications [158,159,160]. Second, the autonomous nature without human participation will lead to physical hijacking. Security measures may, thus, be incorporated into a vehicle. The 6G networks can assess the situations and transmit vehicle-triggered messages. In addition, new kinds of V2X cyberattacks are conceivable in the CAV ecosystem. Advance CAVs are connected to vehicle manufacturers to continuously monitor software-related updates and send to minimize any predicted air problems.

The security and safety of cars and their occupants may nevertheless be affected by weaknesses in the communication channel or falsifying of the data obtained from the cloud services’ manufacturers. Second, the CAV ecosystem features a complicated supply chain with many third-party service providers, including CSPs, roadside equipment (RSE), cloud service providers, and regulators. As a result, it is not easy to enable a uniform standard of safety standards and interoperability.

Finally, the problem of confidentiality may emerge when CAVs gather information on sensor data, travel routes, and their passengers and owners. Such data are hypothetical to malevolent aggressors. Therefore, the CAV Security Framework (NIST) should ensure device security, data privacy, and security according to the National Institute of Standards and Technology (NIST). In particular, when public transit modes such as planes, trains, and buses are being utilized, protecting the personal privacy of 6G services is required. Therefore, CAV security frameworks must consider security convergence by merging physical and cyber safety and the concept of confidentiality by design [161,162,163].

### 6.5. Industry 5.0

Human collaboration with robots and intelligent technology has been identified as the next industrial revolution breakthrough in Industry 5.0 [164]. 6G is crucial for the automated industrial environment’s advancement. Due to high-security threats, Industry 5.0 apps must satisfy basic security essentials such as integrity, availability, authentication, and auditing. For Industry 5.0 security methods, issues such as lower operational costs, a more comprehensive range of devices, and greater scalability must be considered. Since controlling instructions and monitoring data will be delivered across 6G networks, they will be responsible for data security and integrity protection [165] in Industry 5.0. The 6G era also includes methods and systems for restricting access to sensitive resources such as intellectual property connected with Industry 5.0 that are highly scalable and automated.

### 6.6. Smart Grid 2.0

Grid networks are becoming more innovative as intelligent devices, and advanced data analytics methods are developed, moving from Smart grid 1.0 to Smart grid 2.0. Smart grid 2.0 introduces automated smart meter data analysis, line loss analysis, intelligent dynamic pricing, and automation and management of grid distribution. Smart grid 2.0 has self-healing and self-organized capabilities. It does not depend on an external electric power supply [166]. Therefore, it is critical to provide network information and security in smart grid 2.0 to guarantee privacy, reliability, and availability. The most prevalent security weaknesses are physical attacks, software-related threats, threats against control components, and attacks using artificial intelligence/machine learning [167].

Critical services and components such as control elements (SCADA), data access points, and cyber–physical Emergency Management Systems (EMS) [168], as well as billing, metering, and sharing of information, are broadly prone to these attacks. Additionally, improving trust management of a trading mechanism is crucial for Smart grid 2.0. One of the main characteristics of Smart grid 2.0 is the peer-to-peer trading of energy [169]. Because of these attacks, a third party should build trust with as little involvement as possible.

### 6.7. Digital Healthcare

Digital healthcare is growing in new ways. Intelligence healthcare powered by AI will be advanced through many novel methodologies within the next few years. In addition, the aging population may result in a more significant focus on digital health than has previously been recognized. Body Area Networks (BANs) equipped with intelligent embedded systems advance individualized management and health monitoring. These tailored BANs can gather health data from various sensors, share it dynamically, and interact with network services [170]. 6G will likely become the central communication platform for intelligent future healthcare services. Thus, in the 6G future, device authentication, secure communication, and access control for billions of tiny health devices will be security obstacles to solve.

Data security and ethical usage of electronic records will be critical in the future healthcare system. As previously mentioned, artificial intelligence is needed to control many IoMT devices and analyze data related to health. AI models, in particular, should follow an objective, ethical standards for data collection and model training [171]. 6G networks should protect patient information and data privacy and security as the primary communication backbone for future healthcare systems.

### 6.8. Digital Twins (a Digital Reflection of the Real World)

Communication and AI technologies’ advancement, objects and processes will be digitally duplicated. Intelligent mapping of human-to-human and thing-to-thing interactions in the digital environment will occur. Implementing complex algorithm models, the digital world may simulate, predict, validate, and control physical processes or objects. Then, provide the best answer to physical world problems. The 6G era heralds the dawn of the digital twin age. In healthcare, medical systems may use digital twin data to assist in diagnosis and therapy selection. In the industrial area, digital optimization of product design may help decrease costs and increase productivity. Physical and digital contact and cognitive intelligence networks can rapidly adapt to complex and dynamic settings, enabling autonomy throughout the lifecycle of operation and maintenance, from planning through the building, optimization, monitoring, and self-healing. However, this will complicate the design and capabilities of 6G networks. For example, 6G networks must enable trillion-level devices’ connections and millisecond latencies to detect any variation in the physical environment in real time. Data quality must be maintained via the use of data models and standard interfaces that are capable of self-correction and creation. 6G networks must enable data storage, collecting, training, processing, and modeling in distributed and centralized architectures to satisfy data privacy and security standards.

Additionally, Tbps or higher transmission rates will be needed to satisfy the volume of data required for accurate simulation, verification, and modeling. Digital devices may also be created in a centralized or distributed manner as needed via rapid iterative optimization and decision making. The digital twin is an automation and novel industrial control system identified as a critical application for 6G networks. The digital twin connects the physical and virtual worlds by gathering data from IoT devices associated with the physical systems in real time. Distributed servers will store the collected data situated across the network. Next, the assets’ virtual representation will analyze and evaluate these data. The simulation findings are then used to apply the settings to real-world systems. Integrating data in real and virtual presentations allows performance optimization of physical assets.

Automation, industry 5.0, utility management, healthcare, and contracts are other use cases for digital twins. The primary security risk associated with a digital replicated system is that an attacker may modify, replay, intercept, and replay any communications between digital and physical environments. Therefore, when broad digital twin replicated systems adoption occurs, 6G must allow high secured channels. Another type of attack related to the digital twin systems is that an attacker may manipulate or modify IoT data, thus violating the system’s privacy. Therefore, it is essential when 6G enables a digital twin system to employ IoT protection measures for privacy and data integrity. Blockchain may be an excellent candidate to provide these capabilities in 6G networks [172].

### 6.9. Brain–Computer Interactions (BCI)

The fundamental idea underlying BCI is to link the brain with devices. The devices might be inside the human (i.e., the visual cortex) or externally (i.e., an artificial limb). The BCI process consists of four phases: signs capture, extraction of features, translation of features, and final reporting. The primary applications of BCI are associated with the health care sector, mainly to allow disabled persons to manage the supportive equipment. BCI communication is threatened by different types of attacks that limit the applicability of these applications and may threaten the patient’s life sometimes while using BCI in health applications. BCI attacks can be divided into brain signal generation attacks, data processing attacks, and data acquisition attacks.

Adversarial attacks and misleading stimuli attacks are defined as brain signaling attacks. Adversarial attacks present an ML system with intentionally crafted inputs to disrupt its regular operation and output. On the other hand, misleading stimuli target the presentation of incorrect sensory inputs to users to elicit a particular brain response.

**Figure 10:**
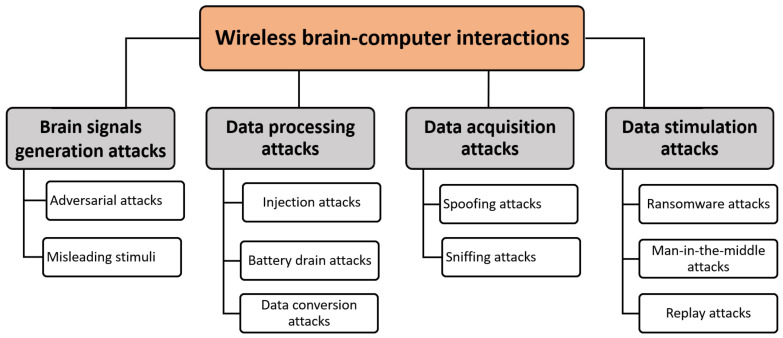
The wireless brain-computer interaction attacks and threats.

Battery drain attacks, data conversion attacks, and injection attacks are examples of data processing attacks. Battery threats deplete a device’s battery, reducing performance or rendering it unusable. Furthermore, injection attacks provide interpretations with input containing specific elements that can modify how the inputs are evaluated, benefiting from an absence of input validation. Sniffing, replay, and spoofing attacks are data acquisition and stimulation attacks that threaten BCI security. BCI attacks are shown in Figure 10.

Chen et al. [173] introduced a new BCI technique in 2015, aimed to enhance orthography using brain signals. BCI was expected to find much applicability with the emergence of 6G networks. However, comparable to XR, BCI applications were highly physically sensitive and required a Quality of Physical Experience Guarantee (QoPE). Wireless BCI technology mainly focuses on data security in terms of misbehavior and encryption.

Mccullagh et al. [174] highlighted that data protection in wireless BCI was one of the primary challenges. Ramadan et al. [175] proposed some malware applications to access neurologically confidential information. Švogor et al. [176] suggested an accessing technique using a password that requires the user to reach a particular psychological condition to resist reply threats. In addition to improvements in the remarkable capabilities of wireless BCI, the security approach of Karthikeyan et al. [177] increases the level of security. Table 3 summarizes the wireless brain communication attacks and their threat impact on 6G network security.

### 6.10. Distributed Ledger Applications

The technology of blockchain exchanges information with all included parties, and it is expected to use blockchain to share spectrum and data, improving the 6G networks’ security. Li et al. [178] mentioned three categories of attacks: (1) the vulnerable attack, (2) the privacy leakage attack, and (3) the double-spending attack. They also provided solutions based on blockchain in 6G networks, such as cryptography algorithms and incentive schemes.

Dai et al. [179] remarked that specific blockchains provide poor security, such as privately-owned blockchains and high-level security, e.g., consortium blockchains. The high-level security blockchains are available for secure resources transactions. Table 4 summarizes the security challenges and requirements of the mentioned 6G applications. In addition, Table 5 introduces the 6G-related works of upcoming 6G applications and the fundamental contributions of each technology. In Table 5, we can observe the most common attacks on the promising 6G applications that relate to access control, authentication, malicious behaviors, and privacy issues. Table 5 also summarizes the possible solutions presented by some related work that propose some security approaches using different AI and ML techniques to detect prevent malicious attacks. All current solutions target the strengthening of security in different aspects, starting from the user end to the application and devices.

## 7. Conclusions

This paper introduces an intensive study on security challenges and requirements for the 6G network. It shows the evolution of security in legacy wireless networks, starting from the 1G network to the upcoming 6G network. In this paper, we proposed the 6G network vision and research directions in academia and industry. We also proposed a 6G security architecture and the new expected security functions. We covered the different physical layer technologies in 6G networks by investigating the possible attacks and proposed solutions. The expected innovative 6G system includes AI technologies to enhance security and increase network protection. Thus, the paper discusses the security architecture of the 6G network based on AI/ML technologies. The layers of security architecture include the intelligent sensing layer, intelligent edge layer, intelligent control layer, and intelligent application layer. Each layer supports various functions and introduces some attacks. Several security issues of the physical layer have been addressed, such as molecular communication, THz communication, and VLC communication. Most of the new 6G technologies pose significant security and privacy threats. These leading technologies have been highlighted, clarifying their security challenges and attacks and security prevention solutions. Every new generation of network technology introduces innovative and creative applications. 6G can use specific apps from earlier radio generations. 6G is quickly establishing itself as the network enabler for several other new applications that will fundamentally alter human civilization in the 2030s and beyond. Many apps and services have highly demanding performance and incredibly severe specific security because of the high communication requirements and needs of 6G applications. The paper presents different security challenges and necessities for several 6G applications such as unmanned ariel vehicles, holographic, extended reality, industry 5.0, Smart grid 2.0, health care, and brain-computer interactions. Potential 6G developments and difficulties for various 6G applications are also discussed. We intend to investigate the different attacks on the 6G network with greater depth in the future. Finding a solution for protecting 6G is a critical issue that will need to be researched in the future.

## Figures and Tables

**Table 1:** Security and privacy issues in earlier mobile networks.

Mobile Networks	Supported Services and Functions	Security and Privacy Issues
1G	Deliver voice communications services Uses analog modulation techniques, lacks a specified wireless standard	Unencrypted nature of telephone services Unauthorized access and eavesdropping attacks Cloning attacks
2G	Enable voice and short messaging services Anonymity is achieved via anonymous identifiers TMSI privacy solution and radio path encryption	Unauthorized access One-way authentication issue IMSI-catcher attacks Traceability attacks Eavesdropping attacks End to end encryption problem
3G	Provide internet access Advanced services such as TV streaming, internet browsing Air interface security and user authentication 3GPP supports various privacy considerations for 3G networks include securely locating, identifying	Two-way authentication Authentication server attacks Integrity threats, Unauthorized data access, Denial of Service (dos) attacks Unauthorized service access AKA sniffing attacks
4G	Handle complex applications such as High-Definition Television (HD TV) Support diversity of intelligent mobile terminals 4G networks offered up to 1 Gbit per second for downlink transmission 500 Mbit per second for uplink communication	Tampering hardware platforms Viruses and operating system attacks Medium Access Control (MAC) layer vulnerabilities Eavesdropping and replay attacks Data integrity attacks Unauthorized access attacks Authentication issues
5G	Connecting higher number of growing devices Delivering higher quality services to all network entities Enhanced Ultra-Reliable, Low Latency Communication (ERLLC) Software-Defined Networking (SDN) and Network Functions Virtualization (NFV) Support high requirements to ensure service and resources availability and continuity	DoS or resource attacks Hiding of active and passive eavesdropping using large MIMOs SDN threats and rogue applications NFV services security problems 5G-AKA attacks and issues IMSI-catcher attacks Voice IP attacks Traceability attacks Exploiting information from failure messages

**Table 2:** The 6G technologies, security challenges, and related work basic contributions.

6G Physical Layer Technology	Related Work	Security and Privacy Challenges	Basic Contributions
THZ	Akyildiz et al. [81]	Authentication	They discuss the electromagnetic signatures of THz frequencies that may be employed in physical layer authentication procedures.
	Ma et al. [82]	Malicious behaviors	They claim that an eavesdropper can capture a THz signal by using narrow beams. Moreover, they talk about a means of resisting this type of attack.
VLC	Pathak et al. [91]	Malicious behaviors	They highlighted what the victim’s line of sight should be if the adversary intends to conduct an attack on the current VLC process.
	Ucar et al. [92]	Privacy of communication	They introduced a SecVLC protocol to protect the privacy of data transmissions over vehicular networks.
	Mostafa et al. [93]	Encryption	They proposed a precoding technology that guarantees the efficiency of the physical layer and could improve the security.
	Cho et al. [95]	Malicious behaviors and security of the physical layer	They have proven that there could be a potential degrade in VLC safety by collaborating with eavesdroppers.
Molecular communication	Farsad et al. [96]	Malicious behaviors and authentication problems	An extensive overview of current molecular communication developments.
	Lu et al. [97]	Molecular communication reliability and encryption	To improve the reliability of transferred data inside a molecular communication system, two different codes are used for the first time. Both codes are Euclidean-Geometry Parity-Check (EG-LDPC) and cyclic-Reed-Muller (C-RM) code.
	Loscri et al. [98]	Authentication challenges and different attacks	Offering some initial insights on the issues of MC system privacy and security. Explores numerous ways for attacking molecular medium at various levels.
AI and ML technology	Dang et al. [114]	Authentication	Claim that AI design might support in identifying network problems in the 6G security and provide prevention approaches and protection solutions.
	Zhou et al. [113]	Access control and authentication	Explores AI technologies as well, claimed to detect security risks in advanced computing in greater detail.
	Sattiraju et al. [110]	Authentication	They proposed an efficient learning approach to improve the security of the physical layer in the authentication process.
	Hong et al. [111]	Communication	Presented an antenna design for classification tasks that must be used in communication with the physical layers to prevent any information leakage.
	Nawaz et al. [112]	Encryption	The proposed protection for the communication links in 6G networks using machine learning techniques and quantum encryption solutions.
Quantum communication	Hu et al. [119]	Quantum secret sharing, key management, and security of direct communication	Ensure the proper security of quantum communication. The experiment showed clearly the possibility of direct quantum-safe communication during a noisy and lossy environment. They also reported the first experiment based on a DL04 protocol and the coding for the frequency of a single-photon, which has validated block transmission.
	Zhang et al. [120]	Encryption	They allow the transmission of encrypted messages through a direct channel without using a private key. Providing fundamental steps towards practical quantum secure direct communication (QSDC) for long-distance quantum communication using quantum memory.
	Nawaz et al. [112]	Encryption of secret key	Using machine learning techniques to support key security.
Distributed ledger technology	Ling et al. [130]	Authentication	They proposed a novel network radio access architecture based on blockchain (B-RAN) to develop a secure efficient decentralized mechanism to manage authentication procedures and network access among many network components.
	Kotobi et al. [131]	Access control	They presented a way to enhance media access protocol and cognitive radio safety by leveraging the blockchain to obtain access to the unused licensed spectrum.
	Ferraro et al. [133]	Access control	They provide a framework for the application of Distributed Ledger Technology (DLTs) as a social compliance control mechanism in smart city environments that can improve the security against double-spending attacks.

**Table 3:** The wireless brain communication attacks and their threat impact on 6G network security.

BCI Attacks		Threat Impact
Brain signal generation attacks	Adversarial attacks	Giving a machine learning system the wrong information to make it malfunction and generate inaccurate results.
	Misleading Stimuli attacks	Users are subjected to harmful sensory stimuli with the goal of inducing a certain brain reaction.
Data acquisition attacks	Sniffing attacks	Obtaining sensitive information through a communication link. When data are not protected, hackers may access and investigate anything, even the details of communication.
	Spoofing attacks	This is conducted by pretending to be a communication entity. IP and MAC spoofing are two common spoofing techniques in network communications.
Data processing attacks	Injection attacks	Using the fact that input is not validated, provide an interpreter with input having multiple components that may alter how it is handled.
	Battery drain attacks	Batteries may run out, and if they do, the device can no longer be utilized.
	Data conversion attacks	It is possible to tamper with both neurological data collecting and stimulation.
Data stimulation attacks	Man-in-the-middle attacks	Communication between two entities is adjusted such that the extremes believe they are speaking directly.
	Replay attacks	Sending the same data repeatedly to disrupt the network owing to lack of input verification
	Ransomware attacks	Encrypt user data and then demand a monetary ransom to be able to decode it is the goal.

**Table 4:** The 6G applications security challenges and the basic security requirements.

6G Application	Security Challenges	Security Requirements
UAV based mobility	High altitude and High mobility Limited energy Diversity of devices Terrorist attacks Physical tampering	Diversity of devices Real-time operations with reduced operational cost High scalability End to End security system design
Telepresence holography	Limited resources Limited energy End to end security system design	High privacy Real-time operation Preventing terrorist attacks
Extended reality	Lack of security standards Physical tampering attacks Limited resources	Edge security Lightweight privacy Real-time operation
Connected Autonomous Vehicles (CAV)	High mobility Physical attacks Privacy challenges Lightweight end to end security Diversity of devices Dynamic security solutions	Lightweight authentication Ultra-Privacy-preserving Proactive security Real-time resistance against attacks Low computation and communication
Industry 5.0	Denial of Service Smart Security Smart Factory Supply chain and Extended Systems	Ultra-High privacy Proactive security Lightweight security Confidential information and intellectual property
Smart grid 2.0	Smart grid attacks Aggregation of data Translation between protocols Physical equipment attacks Exploitation	Scalable IoT security and heterogeneity Zero-touch security High privacy Reduced cost Maintaining access
Artificial intelligence in health care	Novel approaches for dynamic security Diversity of devices Trustworthiness Visibility Ethical and legal aspects Extensibility and viability Controlled security tasks	Diversity of devices High privacy Zero-touch security Edge security Domain-specific security
Digital twins	Security of physical model Security of digital model Diversity of devices Privacy-preserving High mobility Isolated security systems	High bandwidth Ultra-privacy Lightweight security Scalability Dynamic security systems Robustness
Wireless brain–computer interactions	Structure design Physical attacks Privacy challenges End to end security systems	Confidentiality Availability Safety Integrity
Distributed ledger applications	Double-spending Majority vulnerability Scalability Quantum computing Transaction privacy leakage	Preventing privacy leakage Preventing double-spending attack

**Table 5:** The security and privacy challenges of 6G application-related work and their contributions.

6G Applications	Related Work	Security and Privacy Challenges	Basic Contributions
Robotics and autonomous systems	Hooper et al. [138]	Malicious Misbehavior	They mentioned WiFi attacks, which an adversary of Tiro may exploit.
	Fotouhi et al. [139]	Malicious Misbehavior	They study drone attacks through eavesdropping, spoofing, hijacking, and DoS attacks.
	Challita et al. [150]	Attacks, security, and privacy issues	They proposed a network-based artificial neural system to provide secured real-time solutions for automated drone applications
	Sanjab et al. [151]	Authentication and access control	They propose a new mathematical model that supports the trustworthiness of autonomous drone systems.
	Sun et al. [152]	Communication	They introduce a novel way of communication that may avoid eavesdropping attempts.
	Kim et al. [153]	Privacy and authorization	They proposed a framework that would protect the privacy of the UAV Network.
	Xu et al. [154]	Privacy and authentication	They propose an (EPTD) protocol for V2X applications.
	Ni et al. [147]	Authentication and Physical attacks	They provide an autonomous approach that enables two-factor authentication. Reducing physical attacks.
	Wang et al. [157]	Malicious Misbehavior	They highlight the autonomous vehicle’s cyberattacks by employing attacks such as brute force and capturing of packets.
	Tang et al. [106]	Authentication	They introduce a comprehensive paper survey for several machine learning approaches that could be used to improve the 6G security.
Blockchain and distributed ledger technologies	Li et al. [137]	Malicious Misbehavior, Encryption	They provide three categories of threats of harmful behaviors that affect blockchain-based solutions in 6G networks.
	Dai et al. [179]	Authentication and privacy	They remark that privately-owned blockchains are of poor security, and consortium blockchains are of high-security level.
Multi-sensory XR applications	Chen et al. [143]	Malicious behaviors and communication attacks	They observe that sensitive and confidential data can still be disclosed due to some attacks. They claim that the reliability and security of a network are satisfied through solving the 6G network dynamics.
	Hamamreh et al. [144]	Malicious behaviors and attacks	They proposed a method for intercepting and improving security against URLLC eavesdropping attacks.
	Al-Eryani et al. [145]	Access control	They developed the multi-access approach DOMA for multi-sensory XR solutions to extend massive devices’ capability to simultaneously access the 6G networks that could enhance security and reliability.
	Dang et al. [114]	Privacy and secrecy of eMBB applications	They provide details and consideration of privacy, security, and secrecy of eMBB.
	Yamakami et al. [147]	Privacy and authentication issues	They propose a three-dimensional solution to the attacks posed to privacy in the XR solutions.
	Pilz et al. [148]	Privacy	They prove that XR-sensory applications can manage services to improve privacy and security.
Wireless brain–computer interactions	Mccullagh et al. [174]	Encryption	They highlight that data protection in wireless BCI is one of the primary challenges.
	Ramadan et al. [175]	Malicious behaviors	They provide malware applications to obtain access to the sensitive neurological information.
	Švogor et al. [176]	Encryption and Malicious behaviors	They have suggested a technique using a password that needs the user to reach a particular psychological condition to resist reply threats.
	Karthikeyan et al. [177]	Access control	Proposing a security approach for BCI that increases security.
